# Marine fungi showing multifunctional activity against human pathogenic microbes and cancer

**DOI:** 10.1371/journal.pone.0276926

**Published:** 2022-11-28

**Authors:** Fuad Ameen, Saleh AlNAdhari, Ali A. Al-Homaidan

**Affiliations:** 1 Department of Botany and Microbiology, College of Science, King Saud University, Riyadh, Saudi Arabia; 2 Deanship of Scientific Research, King Saud University, Riyadh, Saudi Arabia; VIT University, INDIA

## Abstract

Multifunctional drugs have shown great promise in biomedicine. Organisms with antimicrobial and anticancer activity in combination with antioxidant activity need further research. The Red Sea and the Arabian Gulf coasts were randomly sampled to find fungi with multifunctional activity. One hundred strains (98 fungi and 2 lichenized forms) were isolated from 15 locations. One-third of the isolates inhibited clinical bacterial (*Staphylococcus aureus*, *Bacillus subtilis*, *Vibrio cholerae*, *Salmonella typhi*, *S*. *paratyphi*) and fungal pathogens (*Talaromycets marneffei*, *Malassezia globose*, *Cryptococcus neoformans*, *Candida albicans*, *Aspergillus fumigatus*) and four cancer cell lines (Hep G2 liver, A-549 lung, A-431skin, MCF 7 breast cancer). Bacterial and cancer inhibition was often accompanied by a high antioxidant activity, as indicated by the principal component analysis (PCA). PCA also indicated that fungal and bacterial pathogens appeared to be inhibited mostly by different marine fungal isolates. Strains with multifunctional activity were found more from the Rea Sea than from the Arabian Gulf coasts. The highest potential for multifunctional drugs were observed for *Acremonium* sp., *Acrocalymma* sp., *Acrocalymma africana*, *Acrocalymma medicaginis* (activity reported for the first time), *Aspergillus* sp. *Cladosporium oxysporum*, *Emericellopsis alkaline*, *Microdochium* sp., and *Phomopsis glabrae*. Lung, skin, and breast cancers were inhibited 85%–97% by *Acremonium* sp, while most of the isolates showed low inhibition (ca 20%). The highest antifungal activity was observed for *Acremonium* sp., *Diaporthe hubeiensis*, *Lasiodiplodia theobromae*, and *Nannizia gypsea*. One *Acremonium* sp. is of particular interest to offer a multifunctional drug; it displayed both antifungal and antibacterial activity combined with high antioxidant activity (DPPH scavenging 97%). *A*. *medicaginis* displayed combined antibacterial, anticancer, and antioxidant activity being of high interest. Several genera and some species included strains with both high and low biological activities pointing out the need to study several isolates to find the most efficient strains for biomedical applications.

## 1. Introduction

Microbial metabolites are continuously studied to solve the problem of drug resistant microbes and cancer. High expectations have been set for novel multifunctional drugs due to their high efficacy [1, 2]. Multifunctional metabolites are offered by plants, bacteria, and fungi [3, 4] of which metabolic activity is generally high in extreme environmental conditions [5]. High metabolic activities of fungi have been observed, for instance, in desert soils [6]. Marine habitat is another extreme environment studied as a source of biologically active fungi, as reviewed several times [7–11] recently.

The potential of marine fungi in biomedicine is diverse. Several marine fungal species have been shown to inhibit cancer cell growth [12–14]. The enormous potential of marine fungi to produce antibiotic compounds was reviewed recently [15, 16]. A total of 133 anti-inflammatory compounds produced by marine fungi have been reported [17]. Antifungal compounds produced by marine fungi have also been reported, although much less than antibacterial compounds [9]. Even viruses are inhibited by marine fungal metabolites [18].

Most reports show one or two biological activities at a time. However, recent advances in biomedicine support a new efficient treatment strategy; to combine antioxidants with other biomedicines [19, 20]. Therefore, the organisms offering antioxidant activity in combination with antimicrobial activity and/or cytotoxicity are of great interest. Research on organisms offering these multifunctional drugs is in its infancy and more research on potential organisms producing metabolites that could be used as novel multifunctional drugs is needed.

We aimed to find marine fungi that have high activity against pathogenic bacteria, fungi, and cancer cells in combination with high antioxidant activity. These isolates would offer multifunctional drugs for further biomedical studies. The coast of the two northernmost tropical seas, namely the Red Sea and the Arabian Gulf were sampled. Both seas provide harsh conditions. The Red Sea is one of the most saline and warmest waterbodies, and the Arabian Gulf is the hottest sea in the world [21]. Marine fungi were isolated from 15 different locations on the coasts of the seas around the Arabian Peninsula and studied for their antioxidant activity as well as their activities against both bacterial and fungal pathogens and cancer cells.

## 2. Materials and methods

### 2.1 Sample collection sites and sampling

**Fig 1:**
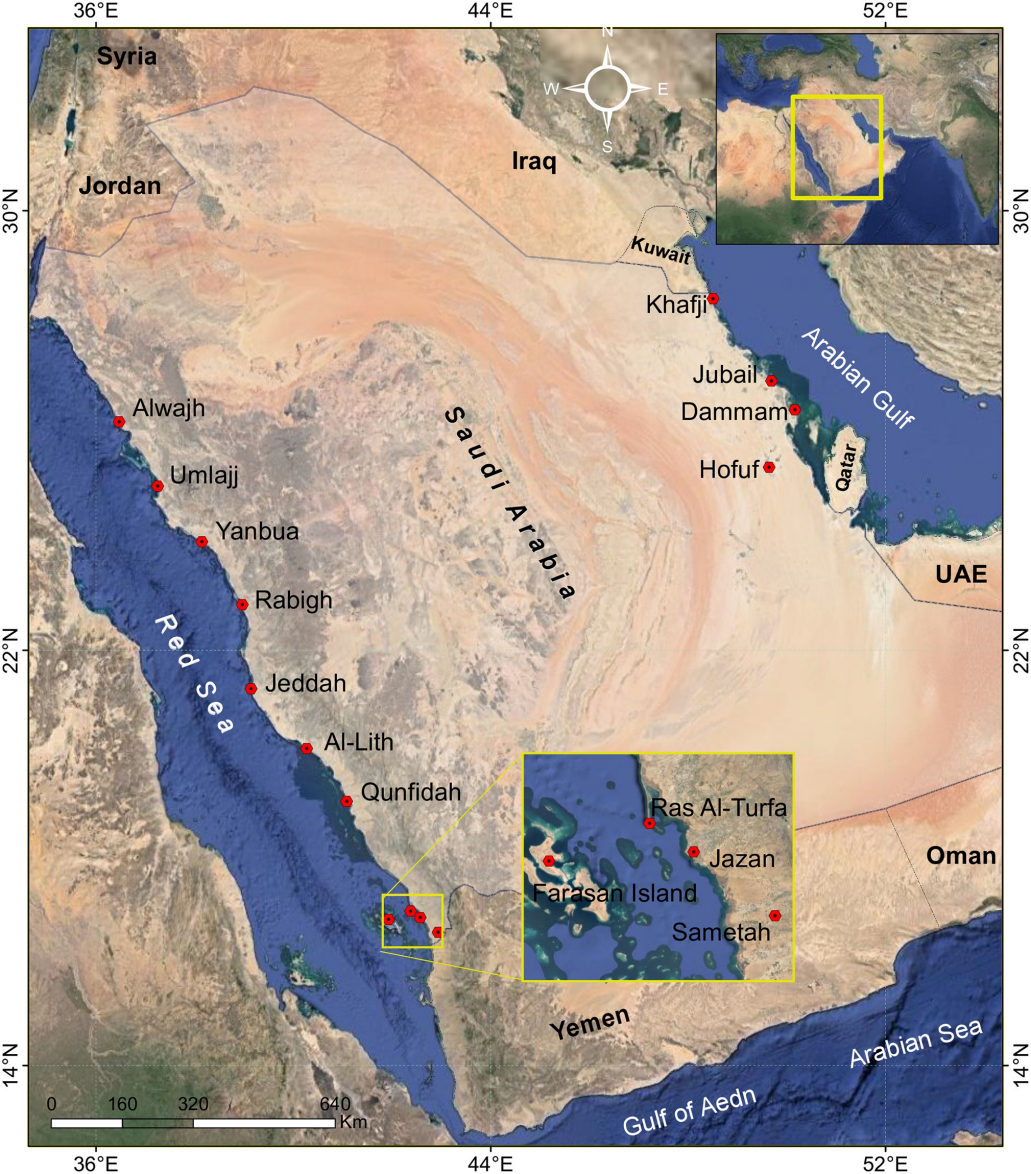
Location map of the study area (The map is modified from Google maps).

Fifteen sites on the coasts of the Arabian Gulf (4 sites) and the Red sea (11 sites) were sampled at low tide. The sites were in Sametah (S), Jazan (S), Ras Al-Turfa (S), Farasan island (S), Qunfidah (S), AlLith (S), Jeddah (W), Rabigh (W), Yanbue (W), Umlajj (N), Alwajh (N), Hofuf (E), Dammam (E), Jubail (E), and Khafji (E) (Fig 1). One water sample (50 mL) was collected from each site, around 450 m away from the shoreline at the depth of 20 cm into a sterilized amber coloured container and transported to the laboratory of Botany and Microbiology, College of Science, King Saud University, Saudi Arabia in November 2021.

The water temperature ranges on the Red Sea coasts between 23°C and 30°C, salinity between 36 PSU and 38 PSU and the pH is 8.1 [22]. The respective values in the Arabian Gulf are 27°C- 33°C, 39.5–42 PSU and 8.2.

### 2.2 Isolation of fungi and pure culture preparation

The seawater samples were serially diluted (10^4^ or 10^5^), and 1 mL was spread on potato dextrose agar (PDA) plates, including antibiotic chloramphenicol 200 μg/L. Plates were incubated at 25°C for 7 days in a laboratory incubator (Remi Lab World). Individual colonies (isolates) were selected based on their morphological characteristics. The selected isolates were sub-cultured on PDA plates and preserved for further studies. Fungal mycelia were transferred into sterile Eppendorf tubes containing 1 mL, 30% (v/v) sterile glycerol and incubated at 28°C for 5 days and then stored at -20°C.

### 2.3 Molecular identification of fungi

The DNA extraction was carried out as described by Ameen et al. [23]. Fungal mycelia were collected into a 2 mL centrifugation tube containing 500 μL of extraction buffer (25 mM EDTA, 0.5% SDS 200 mM, 250 mM NaCl, Tris-HCl, pH 7.5) and centrifuged at 13,000 g for 1 min. The supernatant was transferred into a fresh centrifugation tube followed by the addition of an equal volume of an ice-cold phenol: chloroform mixture (1:1) and centrifuged at 13,000 g for 2 min. The supernatant was collected into another fresh tube with 300 μL of chloroform, then centrifuged at 13,000 g for 2 min (repeated again) and then the supernatant was transferred into a fresh tube with 300 μL ice-cold isopropanol, gently mixed and kept in water bath at 80°C for 30 min. The mixture was then centrifuged at 13,000 g for 5 min, the resultant pellet was collected and washed with 70% ice-cold ethanol, and resuspended in 1 mL sterile water. The yield and quality of the DNA were assessed by agarose gel electrophoresis.

The DNA was amplified using ITS1 (5′-TCCGTAGGTGAACCTGCGG-3′) and ITS4 (5′-TCCTCCGCTTATTGATATGC-3′) primers [24] using 5 min initial denaturation (95° C), 1 min denaturation for 35 cycles (94°C), 30 s annealing (55°C), 2 min extension (72° C), and 10 min final extension (72°C). The sequencing was completed using BigDye terminator sequence kit (Applied Biosystems) and the sequence identification was carried out using NCBI (The National Center for Biotechnology Information, https://www.ncbi.nlm.nih.gov/) databases (BLAST software). The alignments were completed with T-Coffee algorithm (https://www.ebi.ac.uk/, EMBL-EBI, Cambridgeshire, UK). The phylogenetic analysis was carried out using the neighbor-joining method in the MEGA 5.2 program. The sequences were deposited to GenBank. The purified marine fungi are preserved at the university storage.

### 2.4 Metabolite extraction

Fungal metabolites (crude extracts) were collected as described by Ameen et al. [23]. Fungi were first inoculated into conical flasks (2 L) with PDB and incubated for 30 days at 28°C in a static condition. Culture broth was filtrated using Whatman No.1 filter paper and the filtrate was extracted with an equal volume of ethyl acetate. Then, the solution was evaporated using a rotary evaporator (Lab Tech) and dissolved in methanol/DMSO (100 μg/μL). The extract was membrane filtered (0.22 μm) for further studies. All analyses were carried out as three replicates.

### 2.5 Antimicrobial activity

The antibacterial test was carried out using a modified Bauer- Kirby method [25]. Five clinical human bacterial pathogens, *Staphylococcus aureus* (ATCC 6538), *Bacillus subtilis* (ATCC 6633), *Vibrio cholerae* (ATCC 14033), *Salmonella typhi* (ATCC 6539), and *S*. *paratyphi* (ATCC 9150), the microbial cultures were obtained from reference culture collection, Department of Botany & Microbiology, King Saud University, were grown on Mueller-Hinton (HiMedia) agar plates. The paper discs impregnated with the crude extracts of marine fungi (100 μg/μL) were placed on agar and the inhibition zones were measured (Equation number-1) after incubation in static conditions at 37°C overnight. Antibiotics ampicillin and tetracycline, and fungicides gentamycin and fluconazole (5 μg/mL) were used as positive controls.

The antifungal test was carried out as described by Satika et al. [26]. Five fungal pathogens, namely *Aspergillus fumigatus (ATCC 46645)*, *Cryptococcus neoformans (ATCC 32045)*, *Candida albicans (ATCC 10231)*, *Malassezia globosa (ATCC 4612)*, *and Talaromyces marneffei (ATCC 18224)*, provided by the university above, were used. As above, the plates with the paper discs were incubated in static conditions at 28°C for 5 days.

### 2.6 Antioxidant activity analysis

The total reducing power of the crude extracts was studied as described by Oyaizu [27]. The crude extracts and controls were mixed with 0.5 mL potassium hexacyanoferrate (1%) and 0.5 mL phosphate buffer (0.2 M, pH 6.6) and incubated in a water bath (50°C) for 20 min. Then, 10% of TCA (0.5 mL) was added to end the reaction. 1 mL of the upper portion was collected in a separate tube and 0.1 mL ferric chloride solution (0.1%) and 1 mL distilled water were added. After a 10 min incubation at room temperature, the absorbance was measured at 700 nm. The crude extract without marine fungi were used as the negative control and citric acid as the positive control.

Free radical scavenging activity was studied using DPPH assay (2,2-diphenyl-1-picrylhydrazyl) as described by Gang et al. [28]. Crude extracts and controls were incubated at 37°C in darkness for 30 min and the absorbance was measured at the wavelength of 517 nm. The crude extract without marine fungi were used as the negative control and citric acid as the positive control.

The scavenging percentage was calculated using the following formula.

$$\text{Inhibition}\;\text{percentage}\;\left(\%\right)=\left[\left({\text{OD}}_{\text{Control}}-{\text{OD}}_{\text{Sample}}/{\text{OD}}_{\text{Control}}\right)\right]\times 100$$

where optical density (OD) is the absorbance of the sample.

### 2.7 Cytotoxicity analysis

The cytotoxicity of the crude extracts was studied using the 3-(4, 5-dimethylthiazole-2yl)-2, 5-diphenyl tetrazolium bromide (MTT) assay as described by [29]. Cell lines MCF-7 (breast cancer), Hep G2 (liver cancer), A-431 (skin cancer), and A549 (lung cancer) stored at the cell collection center in Riyadh (ECACC Cell lines, Merck) were used. Cells were maintained in a DMEM medium (Dulbecco’s Modified Eagle’s medium) with 10% FBS where 250 mg/L streptomycin, 100 mg/L penicillin, and 2 mM glutamine were added before the incubation at 37°C in a CO_2_ incubator (5%). The cell concentration of 5× 10^4^ cells/well was used (tissue culture grade 96 wells plate flat bottom) in 100 μL DMEM medium and incubated at 37°C for 24 h in a CO_2_ incubator (5%). After the incubation, the crude extracts and control (without marine fungus) (50 μg/mL) and cisplatin (5 μg/mL) as a positive control were added to the wells and incubated for 48 h. MTT (10 μL, 0.5 mg/mL) was added to each well and incubated for 2 h at 37°C for the removal of PBS (phosphate-buffered saline). The DMSO (dimethyl sulfoxide, 100 μL) was added to each well and the absorbance was measured at 595 nm using an ELISA (enzyme-linked immunosorbent assay) reader. The inhibition percentage was calculated as above.

### 2.8 Data analysis

Principal component analysis using a correlation matrix was carried out for the whole dataset using FactoMineR [30] package in RStudio Desktop version 2022.02.0+443 [31]. The means of the three replicates were used making 100 observations and 16 variables altogether.

## 3. Results

### 3.1 Fungal species

**Fig 2:**
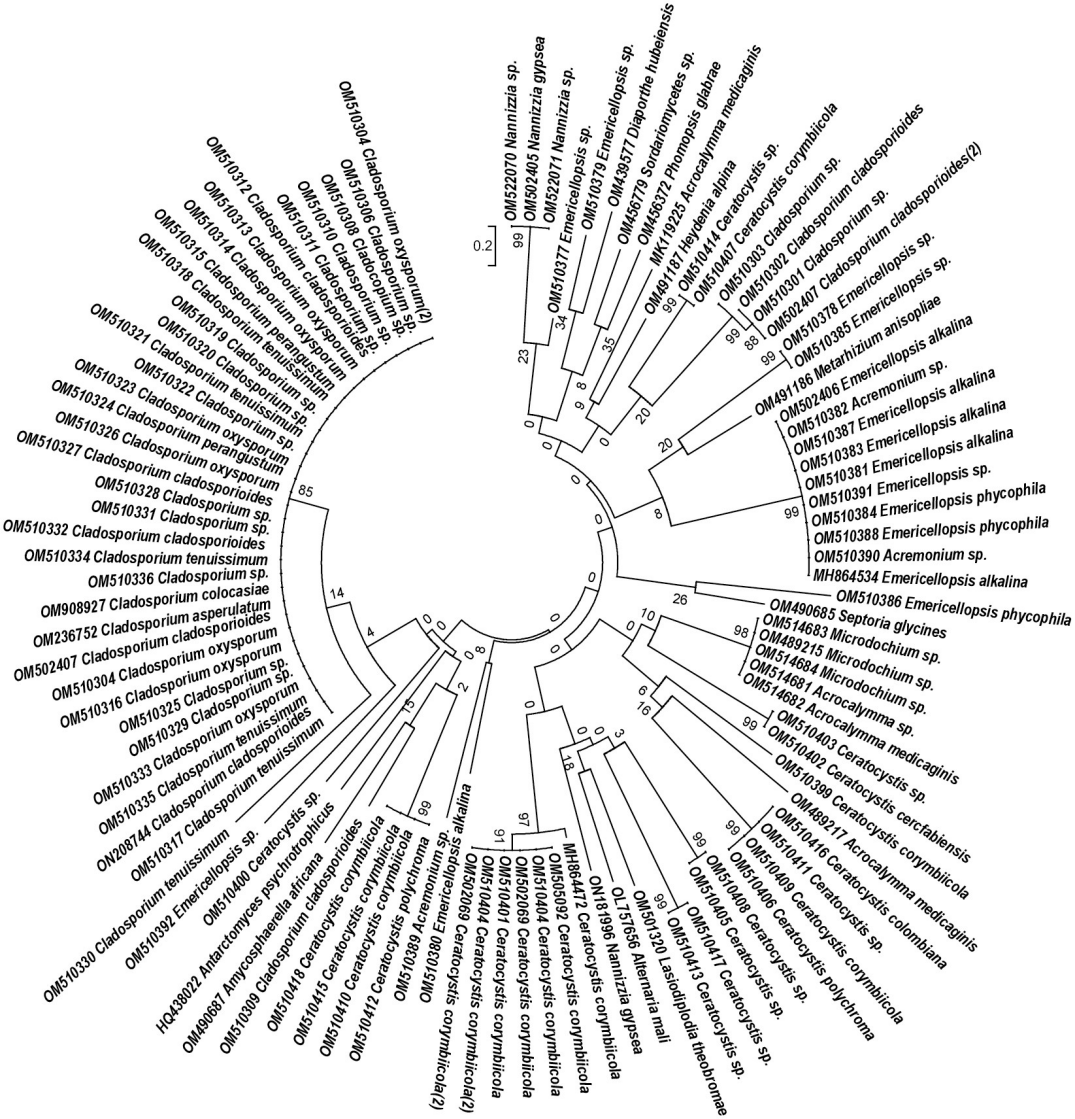
Phylogenetic tree of marine fungi and reference sequence accession numbers constructed by using neighbour joining method in the MEGA 5.2 program.

One hundred isolates, including 31 marine fungal species, were identified (GenBank accession number in Table 1) and their phylogenetic tree is presented (Fig 2). The most common taxon was *Cladosporium* sp., isolated from almost all sites, from 13 out of 15 sampled sites. The most common *Cladosporium* species were *C*. *corybiicola*, *C*. *cladosporioids* and *C*. *tenuissium* isolated from five different sites. While many species were found in many sites, certain species such as *Phomopsis glabrae* and *C*. *perangustum* were found only in one site on the Red Sea coast. *D*. *hubeiensis* and *Heydenia alpina* were found only on the northern Red Sea coast.

**Table 1:** Marine fungi with their National Center for Biotechnology Information (NCBI) accession numbers isolated from different areas in Saudi Arabia.

Species	Name of isolates	GenBank Accession Number	Site	Area
*Acremonium* sp.	JEF1	OM510389	Jeddah	West
*Acremonium* sp.	JEF2	OM510390	Jeddah	West
*Acremonium* sp.	SF3	OM510382	Sametah	South
*Acrocalymma* sp.	JF4	OM514681	Jazan	South
*Acrocalymma africana*	SF5	OM490687	Sametah	South
*Acrocalymma medicaginis*	RAF6	OM514682	Ras Al-Turfa	South
*Aspergillus* sp.	HF7	OM655253	Hofuf	East
*Aspergillus* sp.	DF8	OM655254	Dammam	East
*Aspergillus* sp.	JEF9	OM655255	Jeddah	West
*Buellia* sp.	HF10	OM514685	Hofuf	East
*Buellia lauricassiae*	HF11	OM514686	Hofuf	East
*Buellia lauricassiae*	HF12	OM489216	Hofuf	East
*Ceratocystis* sp.	DF13	OM510400	Dammam	East
*Ceratocystis* sp.	DF14	OM510403	Dammam	East
*Ceratocystis* sp.	JUF15	OM510405	Jubail	East
*Ceratocystis* sp.	KF16	OM510408	Khafji	East
*Ceratocystis* sp.	SF17	OM510411	Sametah	South
*Ceratocystis* sp.	DF18	OM510413	Dammam	East
*Ceratocystis* sp.	DF19	OM510414	Dammam	East
*Ceratocystis* sp.	FF20	OM510417	Farasan island	South
*Ceratocystis cerefabiensis*	QF21	OM510402	Qunfidah	South
*Ceratocystis corybiicola*	SF22	OM505092	Sametah	South
*Ceratocystis corybiicola*	JF23	OM502070	Jazan	South
*Ceratocystis corybiicola*	DF24	OM510399	Dammam	East
*Ceratocystis corybiicola*	DF25	OM510401	Dammam	East
*Ceratocystis corybiicola*	DF26	OM510404	Dammam	East
*Ceratocystis corybiicola*	DF27	OM510407	Dammam	East
*Ceratocystis corybiicola*	DF28	OM510409	Dammam	East
*Ceratocystis corybiicola*	RF29	OM510410	Rabigh	West
*Ceratocystis corybiicola*	YF30	OM510415	Yanbue	West
*Ceratocystis corybiicola*	DF31	OM510416	Dammam	East
*Ceratocystis corybiicola*	DF32	OM510418	Dammam	East
*Ceratocystis polychorma*	AF33	OM510406	AlLith	South
*Ceratocystis polychorma*	JF34	OM510412	Jeddah	West
*Cladosporium* sp.	RF35	OM510301	Rabigh	West
*Cladosporium* sp.	SF36	OM510303	Sametah	South
*Cladosporium* sp.	JF37	OM510306	Jazan	South
*Cladosporium* sp.	RAF38	OM510308	Ras Al-Turfa	South
*Cladosporium* sp.	FF39	OM510310	Farasan island	South
*Cladosporium* sp.	JEF40	OM510311	Jeddah	West
*Cladosporium* sp.	JEF41	OM510319	Jeddah	West
*Cladosporium* sp.	JEF42	OM510320	Jeddah	West
*Cladosporium* sp.	JEF43	OM510322	Jeddah	West
*Cladosporium* sp.	JEF44	OM510328	Jeddah	West
*Cladosporium* sp.	SF45	OM510325	Sametah	South
*Cladosporium* sp.	SF46	OM510329	Sametah	South
*Cladosporium* sp.	HF47	OM510331	Hofuf	East
*Cladosporium cladosporioids*	JF48	OM510302	Jazan	South
*Cladosporium cladosporioids*	RAF49	OM510309	Ras Al-Turfa	South
*Cladosporium cladosporioids*	FF50	OM510312	Farasan island	South
*Cladosporium cladosporioids*	QF51	OM510327	Qunfidah	South
*Cladosporium cladosporioids*	JEF52	OM510332	Jeddah	West
*Cladosporium cladosporioids*	JEF53	OM510304	Jeddah	West
*Cladosporium cladosporioids*	JEF54	OM502407	Jeddah	West
*Cladosporium oxysporium*	JEF55	OM510310	Jeddah	West
*Cladosporium oxysporium*	UF56	OM510314	Umlajj	North
*Cladosporium oxysporium*	JF57	OM510316	Jazan	South
*Cladosporium oxysporium*	JF58	OM510326	Jazan	South
*Cladosporium oxysporium*	JF59	OM510323	Jazan	South
*Cladosporium oxysporium*	JUF60	OM510333	Jubail	East
*Cladosporium perangustum*	JEF61	OM510307	Jeddah	West
*Cladosporium perangustum*	JEF62	OM510315	Jeddah	West
*Cladosporium perangustum*	JEF63	OM510324	Jeddah	West
*Cladosporium tenuissium*	KF64	OM510305	Khafji	East
*Cladosporium tenuissium*	JEF65	OM510317	Jeddah	West
*Cladosporium tenuissium*	UF66	OM510321	Umlajj	North
*Cladosporium tenuissium*	JEF67	OM510330	Jeddah	West
*Cladosporium tenuissium*	FF68	OM510334	Farasan island	South
*Diaporthe hubeiensis*	UF69	OM459577	Umlajj	North
*Emericellopsis* sp.	QF70	OM510377	Qunfidah	South
*Emericellopsis* sp.	AF71	OM510378	AlLith	South
*Emericellopsis* sp.	JF72	OM510379	Jeddah	West
*Emericellopsis* sp.	FF73	OM510392	Farasan island	South
*Emericellopsis* sp.	FF74	OM510385	Farasan island	South
*Emericellopsis* sp.	FF75	OM510391	Farasan island	South
*Emericellopsis alkalina*	FF76	OM502406	Farasan island	South
*Emericellopsis alkalina*	FF77	OM510380	Farasan island	South
*Emericellopsis alkalina*	JF78	OM510381	Jazan	South
*Emericellopsis alkalina*	RAF79	OM510383	Ras Al-Turfa	South
*Emericellopsis alkalina*	FF80	OM510387	Farasan island	South
*Emericellopsis phycophila*	AF81	OM510384	Alwajh	North
*Emericellopsis phycophila*	HF82	OM510386	Hofuf	East
*Emericellopsis phycophila*	DF83	OM510388	Dammam	East
*Fusarium magnifereae*	JEF84	OM487085	Jeddah	West
*Heydenia alpina*	AF85	OM491187	Alwajh	North
*Lasiodiplodia theobrome*	DF86	OM501320	Dammam	East
*Microdochium* sp.	SF87	OM514683	Sametah	South
*Microdochium* sp.	HF88	OM514684	Hofuf	East
*Microdochium* sp.	HF89	OM489215	Hofuf	East
*Metarhizium anisopliae*	SF90	OM491186	Sametah	South
*Nannizzia* sp.	DF91	OM522070	Dammam	East
*Nannizzia* sp.	JUF92	OM522071	Jubail	East
*Nannizzia gypsea*	HF93	OM502405	Hofuf	East
*Phomopsis glabrae*	YF94	OM456372	Yanbue	West
*Sordariyomycetes* sp.	RF95	OM456779	Rabigh	West
*Sordariyomycetes glycines*	SF96	OM490685	Sametah	South

Two isolates of the lichen *Usnea*, which is a symbiosis of algae and fungi, was found from the rocky shores of the sea. The species *Usnea* was obtained by the extraction protocol carried out and the identification of the sequence was obtained from NCBI. The species cultured for the activities measurements was an unidentified mycobiont, which belongs to Ascomycota. The mycobiont is called *Usnea* herein.

### 3.2 PCA

The two first axes of PCA explained 72% of the variation. PC1 explained 50%, and PC2 explained 22% of the variation, the eigenvalues being 8.1 and 3.5, respectively.

**Fig 3:**
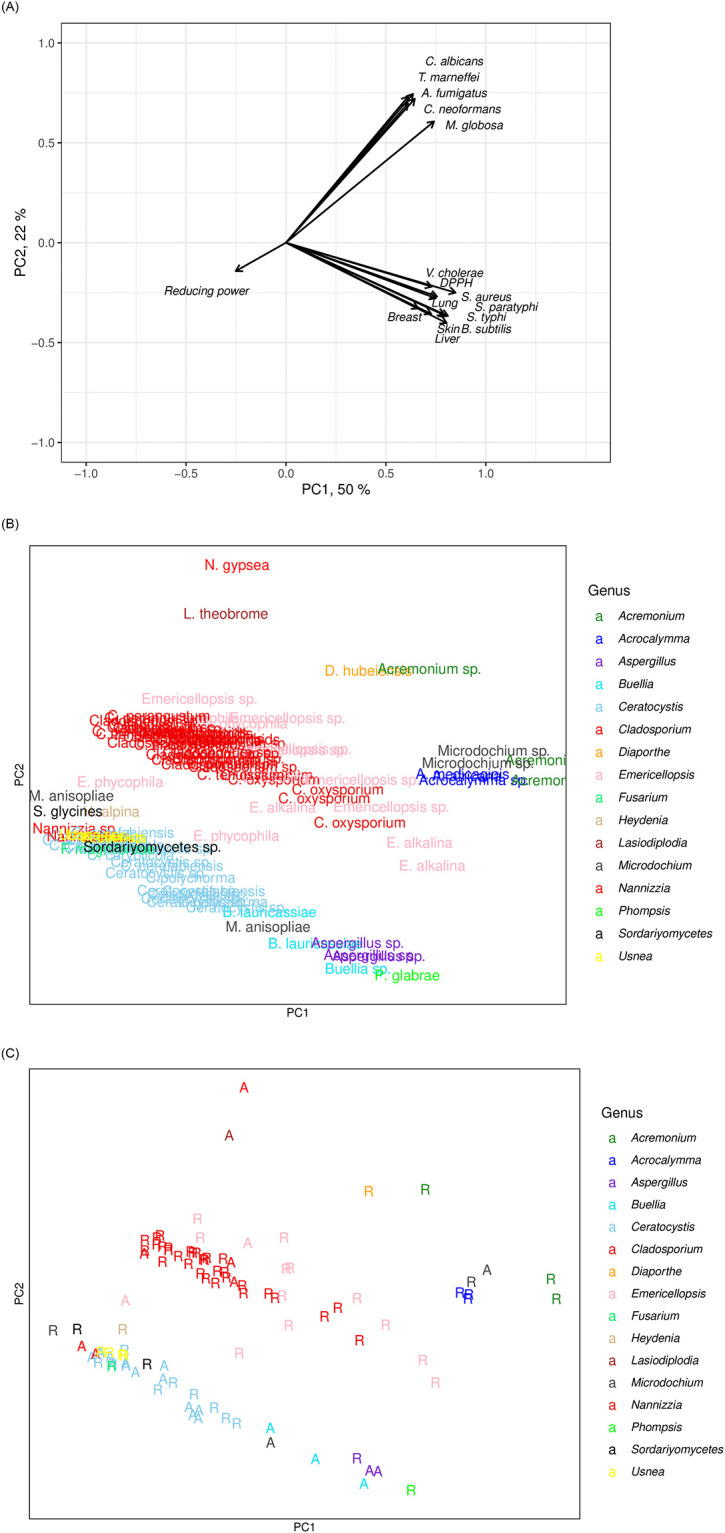
a. Loading’s of variables in PCA where marine fungal metabolites were measured for their inhibitory effect against bacteria, fungi, and cancer and their DPPH scavenging activity and reducing power. b. PCA sample score plot of marine fungal species. c. PCA sample score plot of marine fungal species marked according to their sampling location on the coast of the Red Sea (R) or the Arabian Gulf (A).

The loadings of the PC1-axis were relatively high (> 0.5) for all variables except for reducing power, as indicated by the loadings plot (Fig 3a). The PC1 loadings were highest for DPPH (0.85) and for all four cancer types (0.80–0.78). The inhibition of bacterial pathogens had loadings varying from 0.78 to 0.69. The inhibition of fungal pathogens had slightly lower loadings varying from 0.59 to 0.72. We interpreted that PC1 describes the general biological activity of the marine fungi, the biological activity of the isolate being higher the higher its score on PC1 (Fig 3b).

The loadings plot also indicated that the biological activity variables were separated by PC2, some having positive and some negative PC2 values (Fig 3a). PC2 was interpreted to separate the marine fungi according to their activity against either pathogenic fungi or bacteria. The latter marine isolates also had high cytotoxicity and DPPH scavenging activity, as indicated by their high negative loadings on PC2-axis. However, negative PC2 loadings were relatively low for all variables mentioned (bacteria, cancer, DPPH: 0.22–0.36). PC2 positive loadings of fungi varied between 0.63 to 0.76.

The highest positive PC1 scores (biological activity) were for the marine fungal genera *Acremonium*, *Acrocalymma*, *Microdochium*, *Emericellopsis*, *Phomopsis*, *Aspergillus*, *Diaporthe*, *Buellia*, and *Cladosporium* (Fig 3b). These genera had the 25 highest PC1 scores. The species identified were *A*. *africana*, *A*. *medicaginis*, *E*. *alkalina*, *P*. *glabrae*, *D*. *hubeiensis*, and *C*. *oxysporium*. All of these strains except one *Microdochium* sp. and the two *Aspergillus* sp. were isolated from the Red Sea (Fig 3c). The highest negative PC1 scores indicating no or low biological activities were for the genera *Ceratocystis*, *Cladosporium*, *Sordariyomycetes*, *Emericellopsis*, *Heydenia*, *Fusarium*, *Nannizzia*, and *Microdochium*. These isolates were collected more often from the Arabian Gulf than from the Red Sea (Fig 3c). The sampling sites within the seas were mixed and showed no grouping.

The highest positive scores on PC2 (inhibitors of fungi) were for *Nannizia gypsea*, *Lasiodiplodia theobrome*, *Diaportha hubeiensis*, and *Acremonium* sp. (Fig 3b). The two first with the highest scores were isolated from the Arabian Gulf (Fig 3c). Other isolates having relatively high antifungal activity were *Emericellopsis* sp., *E*. *phycophila*, *Cladosporium perangustum*, *C*. *tenuissium*, and *C*. *cladosporioids*.

The highest negative PC2 scores (inhibitors of bacteria) were for the genera *Phompsis* > *Buellia* > *Aspergillus* > *Microdochium* > *Ceratocystis*. The species identified were *P*. *glabrae*. *B*. *lauricassiae*, *M*. *anisopliae*, and *C*. *polychorma*. *P*. *glabrae* and one *Aspergillus* sp. were isolated from the Red Sea, while the rest of the most efficient mentioned were isolated from the Arabian Gulf. The isolates of the genera *Emericellopsis* and *Microdochium* were either on the negative or positive sites of the PC2-axis.

### 3.3 Biological activities

**Fig 4:**
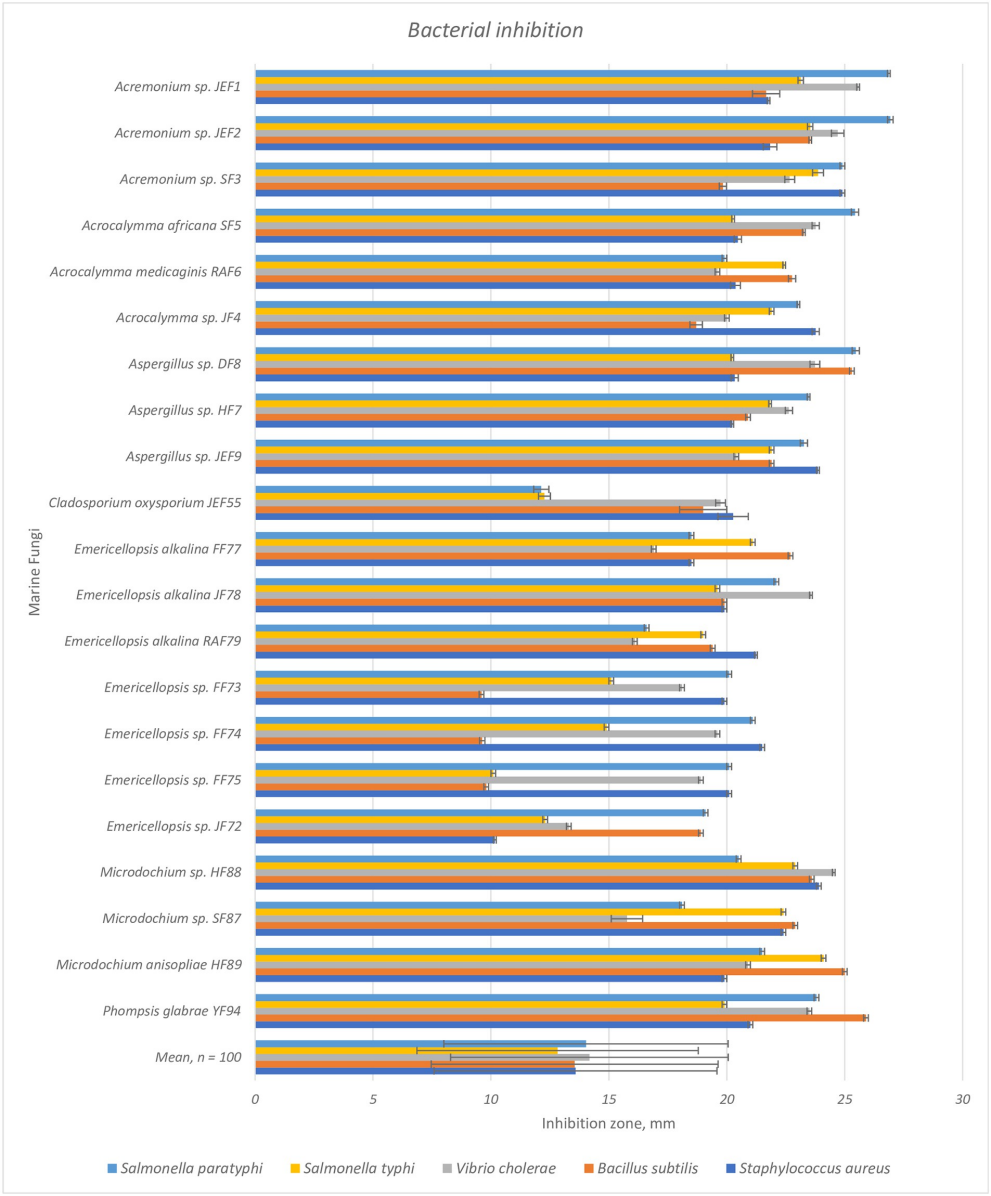
Inhibition (zone of inhibition, mean and error bar for SD) of five pathogenic bacteria (in colors) by the metabolites of the marine fungi isolated from the coasts of the Red Sea and the Arabian Gulf. Mean refers to the mean of all isolates tested. Letters after the species names refer to the isolate code in Table 1.

**Fig 5:**
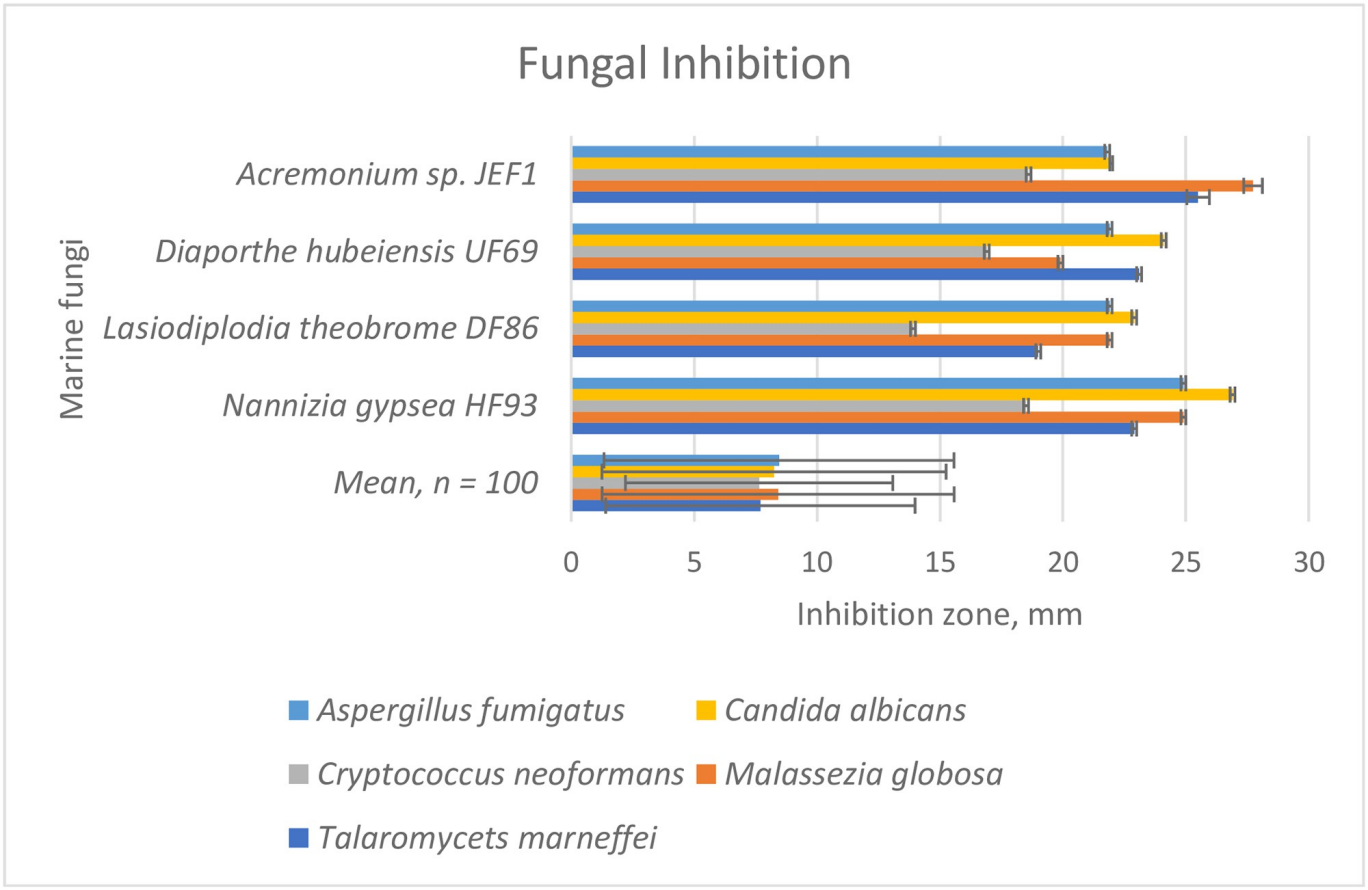
Inhibition (zone of inhibition, mean and error bar for SD) of five pathogenic fungi (in colors) by the metabolites of the marine fungi isolated from the coasts of the Red Sea and the Arabian Gulf. Mean refers to the mean of all isolates tested.

One-third of the extracts (33 out of 100) showed relatively high antibacterial activity (mean inhibition zone ≥ 20 mm), measured as the inhibition zone, when compared to antibiotics used as positive controls (mean ampicillin 16–17 mm, mean tetracycline 13–17 mm). For most of these isolates, the antibacterial activity varied among the five pathogenic bacteria; the isolates did not inhibit all pathogens to the same extent (S1 Table). The combination of high antioxidant activity and antibacterial activity was displayed by 21 isolates (Fig 4). Eleven marine fungal isolates inhibited (mean inhibition zone ≥ 20 mm) at least two fungal pathogens at the same level as the fungicides used (mean gentamycin 20–24 mm, mean fluconazole 20–23) (S2 Table). Antifungal activity of the four most efficient marine fungal isolates, assessed as their zone of inhibition, was two to fourfold compared to the mean of all isolates (Fig 5). Most of the isolates showed only low antifungal activity and some, in practice, no inhibition (S2 Table).

The genera *Acrocalymma*, *Acremonium*, *Aspergillus*, *Buellia*, *Cladosporium*, *Emericellopsis*, *Microdochium*, and *Phomopsis* had the highest cytotoxicity. Lung cancer cell line A549 was inhibited most (97%) by *Acremonium* sp. SF3 (Table 2). Skin cancer A431 and breast cancer cell line MCF7 were inhibited most by *Acremonium* sp. JEF2, 98% and 85%, respectively. *Aspergillus* sp. HF7, *Microdochium* sp. SF87 and *P*. *glabrae* YF94 inhibited most HepG2 liver cancer cell line, 89% each. Most of the isolates showed low inhibition: the mean inhibition of all 100 marine isolates against the four cancer cell lines varied between 18% and 23% (SD 20–28). The positive control cisplatin displayed 85% inhibition.

**Table 2:** Activity (% of inhibition) against four cancer types (mean ± SD, *n* = 3) of the top 19 marine fungal isolates in comparison to the mean of all 100 marine fungal isolates.

Fungi	Isolate	Lung	A549	Skin	A-431	Breast	MCF-7	Liver	Hep G2
Mean ± SD (*n* = 100)		23	± 27	22	± 28	18	± 20	21	±22
*Acrocalymma africana*	SF5	58	± 9	65	± 5	40	± 7	70	± 7
*Acrocalymma medicaginis*	RAF6	48	± 2	76	± 3	52	± 5	66	± 5
*Acremonium* sp.	SF3	97	± 3	90	± 8	80	± 3	67	± 3
*Acremonium* sp.	JEF2	93	± 9	98	± 5	85	± 9	69	± 5
*Aspergillus* sp.	DF8	44	± 2	48	± 5	56	± 2	68	± 4
*Aspergillus* sp.	HF7	37	± 3	55	± 3	62	± 3	89	± 7
*Buellia* sp.	HF10	85	± 9	88	± 9	54	± 6	68	± 5
*Buellia lauricassiae*	HF11	86	± 3	66	± 2	23	± 4	45	± 2
*Cladosporium oxysporium*	JEF55	84	± 5	55	± 8	50	± 7	28	± 8
*Cladosporium oxysporium*	UF56	58	± 5	42	± 4	35	± 8	29	± 4
*Cladosporium oxysporium*	JF57	54	± 3	39	± 7	28	± 4	32	± 7
*Emericellopsis* sp.	JF72	81	± 8	80	± 5	63	± 7	59	± 5
*Emericellopsis* sp.	AF71	78	± 4	86	± 3	52	± 5	60	± 4
*Emericellopsis alkalina*	JF78	93	± 7	90	± 5	55	± 3	62	± 7
*Emericellopsis alkalina*	FF76	86	± 5	80	± 5	45	± 6	52	± 8
*Emericellopsis alkalina*	FF77	82	± 3	87	± 9	50	± 9	58	± 4
*Microdochium* sp.	HF88	88	± 6	68	± 2	35	± 2	56	± 7
*Microdochium* sp.	SF87	83	± 4	45	± 3	52	± 3	89	± 3
*Phomopsis glabrae*	YF94	86	± 7	62	± 9	73	± 9	89	± 9

High antioxidant activity, evaluated as DPPH scavenging activity, in combination with high antimicrobial or anticancer activity was observed for 23 marine fungal isolates (Table 3). These isolates were *Acremonium* sp., *Acrocalymma* sp., *A*. *africana*, *A*. *medicaginis*, *Cladosporium oxysporium*, *Emericellopsis* sp. *E*. *alkalina*, *Microdochium* sp., *M*. *anisopliae*, and *P*. *glabrae*, the DPPH scavenging % varying between 62 ± 5% for *E*. *alkalina* RAF79 and 97 ± 5% for *Acremonium* sp. SF3. The mean of all 100 marine fungi DPPH% was relatively low, 29% with SD of 33%. Antioxidant activity evaluated as reducing power was low for almost all marine fungi. Only six *Ceratocystis* sp. isolates (JUF15, KF16, SF17, DF18, DF19, FF20) showed some reducing power (21–31%).

**Table 3:** Antioxidant activity (DPPH scavenging activity %) of marine fungal isolates (mean ± SD, *n* = 3) with high antibacterial, antifungal, or anticancer activity (referred as colors) in comparison with the mean of all marine fungal isolates.

Marine fungal Isolates	DPPH%	
Mean ± SD (*n* = 100)	29 ± 33	
*Acremonium* sp. JEF1	93 ± 9	
*Acremonium sp*. JEF2	94 ± 7	
*Acremonium* sp. SF3	97 ±10	
*Acrocalymma africana* SF5	83 ± 6	
*Acrocalymma medicaginis* RAF6	86 ± 9	
*Acrocalymma* sp. JF4	88 ±10	
*Aspergillus* sp. DF8	85 ± 8	
*Aspergillus* sp. HF7	96 ± 9	
*Aspergillus* sp. JEF9	87 ± 8	
*Cladosporium oxysporium* JEF55	84 ± 8	
*Diaporthe hubeiensis* UF69	8 ± 2	
*Emericellopsis alkalina* FF76	86 ± 9	
*Emericellopsis alkalina* FF77	93 ±10	
*Emericellopsis alkalina* JF78	82 ± 9	
*Emericellopsis alkalina* RAF79	62 ± 5	
*Emericellopsis* sp. AF71	81 ±10	
*Emericellopsis* sp. FF73	74 ± 7	
*Emericellopsis* sp. FF74	70 ± 7	
*Emericellopsis* sp. FF75	72 ± 5	
*Emericellopsis* sp. JF72	72 ±10	
*Lasiodiplodia theobrome* DF86	6 ± 1	
*Microdochium* sp. HF88	76 ± 7	
*Microdochium* sp. SF87	85 ± 9	
*Microdochium anisopliae* HF89	73 ±10	
*Nannizia gypsea* HF93	2 ± 1	
*Phompsis glabrae* YF94	92 ±12	
Anticancer	Antibacterial	Antifungal
Antibacterial + Anticancer	Antibacterial + Antifungal	

## 4. Discussion

Biomedicine is looking for multifunctional drugs because antioxidants seem to improve the efficiency of the treatments of bacterial infections and cancer [32–36]. In our survey, one hundred marine fungal strains included more than 30 potential strains for biomedical applications showing antibacterial, antifungal, antioxidant, and cytotoxic activities to different extents. We chose 23 strains offering the most promising multifunctional drugs to be developed: the strains displayed a varying combination of activities (Table 3). We also raise four fungal inhibitor strains as significant although they showed no antioxidant activity. The reason is that potential antifungal drugs have been found much less than antibacterial drugs. The most potent fungal inhibitors were *Acremonium* sp., *Diaporthe hubeiensis*, *Lasiodiplodia theobrome*, and *Nannizia gypsea*. The isolate *Acremonium* sp. JEF1 was among the best multifunctional candidate for further studies due to its combined antifungal, antibacterial and antioxidant activity. However, this isolate had relatively low cytotoxic activity while two other *Acremonium* isolates had high cytotoxicity. All three *Acremonium* isolates deserve further studies.

The most remarkable result of the PCA was that the antibacterial activity was accompanied by the capability to inhibit cancer cells and high antioxidant activity assessed as DPPH scavenging activity. The result might be generalizable, and it should be studied further. From the practical point of view, these multifunctional isolates were, however, in the minority. The multifunctional potential was observed for *Acremonium* sp., three *Acrocalymma* sp. including *A*. *africana*, and *A*. *medicaginis*, three *Aspergillus* sp., *Cladosporium oxysporium*, two *E*. *alkalina*, two *Microdochium* sp. and *P*. *glabrae*. No fungal agents have been approved as anticancer drugs so far, maybe because the mechanisms in action are not understood [37]. However, many species have been shown for their potential as anticancer agents (S3 Table) [14, 38, 39], and for instance, several fungal endophytes have been shown to have anticancer activities [40–42].

Another interesting result of the PCA was that marine fungal strains were grouped according to their ability to inhibit bacteria or fungi. Based on this result, it seems that the marine fungal isolates were specialized to inhibit either bacteria or fungi. Only the genera *Acremonium*, *Emericellopsis* and *Microdochium* included both fungal or bacterial inhibitors, and thus, were not specialized. Most published studies deal with either antifungal or antibacterial activities, which is not surprising because the mechanisms of action differ for bacteria and fungi [43]. We found only some studies where both fungi and bacteria were studied. The plant endophytic fungus *Diaporthe schini* metabolites inhibited several bacteria and the fungus *Candida krusei* [44]. The plant root endophytic fungus *Trichoderma hamatum* inhibited both bacterial and fungal plant pathogens [45]. The plant species *Rumex abyssinicusis*, *Tagetes lucida*, and *Lallemantia royleana* have been shown to inhibit both bacteria and fungi [46–48]. *Aloe vera* plant extract inhibited several bacterial species and a few fungal species [49]. When marine fungi metabolites were reviewed by [9], most compounds inhibited bacteria. Out of 170 compounds, 26 were fungal inhibitors, of which only six metabolites inhibited both bacteria and fungi. From that perspective, our findings about fungal inhibitors were of importance. Particularly, we can raise *Acremonium* and *Acrocalymma* isolates that inhibited both bacteria and fungi. However, only one of the isolates had also high antioxidant activity.

The comparison to previous studies reveals that most of the genera we report have already been reported for their biological activities, but seldom for multifunctional activity including antioxidant activity. *Acremonium* and *Emericella* strains isolated from soils, waters, and plants have been shown to produce more than 400 different metabolites with a wide range of biological activities [50–53]. An endophytic *Acremonium* showed antifungal activity against *Pythium* sp. causing root rot [54]. The marine *Acremonium* strain has been reported for antibacterial and also for multifunctional activities including cytotoxicity [55, 56]. *Phomopsis* and *Diaporthe* have been shown to produce more than 300 bioactive metabolites, including cytotoxic and antibacterial compounds [57, 58]. *P*. *glabrae* was among the most active species in our tests. Previously, bioactive polyketides were identified from *Phomopsis* sp. isolated from both marine and terrestrial habitats [13, 59, 60]. *Microdochium* sp. showed antimicrobial activity, as also reported previously [61].

The results of some genera, such as *Ceratocystis* and *Fusarium*, previously reported as potential antimicrobials [62, 63], were not supported by our survey. Some *Cladosporium* isolates collected from all our sampling locations had strong bioactivities. However, most of the commonly found *Cladosporium* isolates showed only weak bioactivities. This is surprising since many *Cladosporium* strains have previously been shown to produce bioactive metabolites [15, 16]. *Cladosporium* sp. has produced almost 300 metabolic compounds with antimicrobial, anticancer, and antioxidant properties [64–66]. Our survey does not raise *Cladosporium* among the most interesting genera in the biomedical field.

The species of *Emericellopsis* are found in different environments and commonly in marine environments. The genus *Emericellopsis* is known for its bioactive properties [50]. In our study, the genus was shown to have antibacterial, antifungal, antioxidant, and anticancer activities. The genus has been of particular interest because it has been reported to produce antimicrobial peptides inhibiting drug-resistant organisms [67]. *Emericellopsis* sp. have been shown to produce several metabolites with antifungal, antibacterial and cytotoxic activities [50, 68]. *Emericellopsis* commonly produce peptides [67] that have been shown for their multifunctional activities [68]. For instance, the peptide emericellipsin A inhibited human pathogenic fungi and bacteria as well as cancer cells [69]. Emericellipsin A affects cell membranes, which has been suggested to be the mechanism behind the multifunctional activities [70].

*A*. *medicaginis* showed bioactivities in our tests. The species was the only one where we found no previous reports about its bioactivities. However, the species appeared to be of particular interest because of its combined antibacterial, anticancer, and antioxidant activity. The fungi *A*. *medicaginis* isolated from the southern Red Sea coast deserve further studies for biomedical applications. Several other isolates such as *Acremonium*, and *D*. *hubeiensis* collected from the Red Sea as well as *L*. *theobrome* and *N*. *gypsea* from the Arabian Gulf coast deserve further studies.

Both seas are hot and saline with no remarkable difference. The greatest difference between the Red Sea and the Arabian Gulf is the depth. While the former reaches 2000 m, the latter reaches only 100 m [22]. The sea level annual cycle and the water outflow differ markedly [71]. Despite some differences, no evident difference between the seas regarding bioactive fungal metabolites was observed. Some of the species such as *C*. *corybiicola* and *C*. *tenuissium* were isolated from both seas. The isolates differed in their activity regardless of the sea. Some isolates of the same species such as *E*. *alkalina* differed in their activity although they were isolated from the same sea. One southern Red Sea isolate of *E*. *alkalina* displayed low activities while four of the isolates displayed high activities. Thus, the sea seemed not to be the explaining factor in general. Many different factors affect the microbial metabolism and more isolates need to be studied. It is known that the environmental conditions modify the pathways of microbial metabolism in complex ways, and therefore, microbes in harsh conditions are of great interest in searching novel drugs and other bioactive molecules [72].

## 5. Conclusion

Marine fungi that were randomly collected from the sea coasts around the Arabian Peninsula showed multifunctional activities potentially valuable for biomedicine. Great differences in the bioactivities of the isolates were observed. Out of one hundred isolates collected, about one-third inhibited pathogenic bacteria, fungi, or cancer cells. All isolates of certain genera, such as *Acremonium*, *Acrocalymma*, and *Aspergillus*, showed some bioactivities. However, in the case of some genera, such as *Cladosporium* and *Ceratocystis*, only a few of the isolates showed remarkable bioactivities. Strains of the same species differed in their activity pointing out the need to test several isolates of the species. It appeared that certain genera were efficient fungal inhibitors while some were bacterial and cancer cell inhibitors. The isolates that had high antibacterial and anticancer activities and were accompanied by high antioxidant activity are particularly interesting in biomedical applications due to their multi-functionality. Further studies on the actual bioactive compounds present in the crude extracts are needed. Our study revealed thirty fungal strains that have the potential to produce multifunctional metabolites and offers them for further studies.

## Supporting information

S1 Table Inhibition (zone mm, mean ± SD, *n* = 3) of four pathogenic bacteria by antibiotics (ampicillin and tetracycline, 5 μg/mL) and marine fungal extracts. Mean ≥ 20 mm against some bacterial pathogens in bold.
(DOCX)

S2 Table Inhibition (zone mm, mean ± SD, *n* = 3) of five pathogenic fungi by fungicides (Gentamycin and Fluconazole, 5 μg/mL) and marine fungal extracts. Mean ≥ 20 mm against some fungal pathogens in bold.
(DOCX)

S3 Table Variable loadings of PCA for different cancer types, bacterial and fungal pathogens and antioxidant activity assays. (DOC)
