# Supplementary material for: Marine fungi showing multifunctional activity against human pathogenic microbes and cancer
Source: PLoS One. 2022 Nov 28;17(11):e0276926. doi: 10.1371/journal.pone.0276926 (PMC9704632; doi:10.1371/journal.pone.0276926)
Supplement: S1 Table — Mean ≥ 20 mm against some bacterial pathogens in bold. (DOCX) [file pone.0276926.s001.docx]

**Marine fungi producing potential multifunctional components against pathogenic microbes and cancer**

Fuad Ameen^1*^, Saleh AlNAdhari^2^ and Ali A. Al-Homaidan^1^

^1^Department of Botany and Microbiology, College of Science, King Saud University, Riyadh 11451, Saudi Arabia

^2^Deanship of Scientific Research, King Saud University, Riyadh 11451, Saudi Arabia

Correspondence to: fuadameen@ksu.edu.sa

**S1 Table**

**S1 Table:** Inhibition (zone mm, mean ± SD, *n* = 3) of four pathogenic bacteria by antibiotics (ampicillin and tetracycline, 5 μg/mL) and marine fungal extracts. Mean ≥ 20 mm against some bacterial pathogens in bold.

| Bacterial inhibitor | *Sstaphylococcus aureus* | *Bacillus subtilis* | *Vibrio cholerae* | *Salmonella typhi* | *Salmonella paratyphi* |
| --- | --- | --- | --- | --- | --- |
| Ampicillin | 17±2 | 16±4 | 16±1 | 16±2 | 16±4 |
| Tetracycline | 16±4 | 13±2 | 15±3 | 16±4 | 17±2 |
| ***Acremonium* sp. (JEF2)** | 22±2 | 22±1 | 26±3 | 23±3 | 27±2 |
| ***Acremonium* sp. (JEF1)** | 22±3 | 23.52 | 25±3 | 24±2 | 27±4 |
| ***Acremonium* sp. (SF3)** | 25±2 | 20±2 | 23±1 | 24±3 | 25±2 |
| ***Acrocalymma* sp. (JF4)** | 24±1 | 19±2 | 20±1 | 22±1 | 23±2 |
| ***A. Africana* (SF5)** | 20±1 | 23±1 | 24±4 | 20±2 | 25±1 |
| ***A. medicaginis* (RAF6)** | 20±1 | 23±3 | 20±1 | 22±2 | 20±2 |
| ***Aspergillus* sp. (HF7)** | 20.2±1 | 21±2 | 22.8±3 | 21.8±2 | 23.4±4 |
| ***Aspergillus* sp. (DF8)** | 20±1 | 25±3 | 24±2 | 20±1 | 25±2 |
| ***Aspergillus* sp. (JEF9)** | 24±2 | 22±3 | 20±2 | 22±3 | 23±2 |
| ***Buellia* sp. (HF10)** | 26±2 | 18±1 | 18.9±3 | 22±4 | 25±3 |
| ***B. lauricassiae* (HF11)** | 25.8±2 | 19.7±2 | 18±2 | 20±2 | 20±2 |
| ***B. lauricassiae* (HF12)** | 21.8±2 | 19±2 | 19.6±2 | 20.9±1 | 21±2 |
| *Ceratocystis* sp. (DF13) | 13.5±2 | 12.9±3 | 8±2 | 10.4±2 | 10.2±3 |
| *Ceratocystis* sp. (DF14) | 15±4 | 7.4±3 | 10±4 | 7.3±2 | 9±4 |
| *Ceratocystis* sp. (JUF15) | 7±2 | 16.4±4 | 19±4 | 18.9±3 | 19±2 |
| *Ceratocystis* sp. (KF16) | 8±4 | 18.2±3 | 19.6±4 | 19.4±4 | 11±2 |
| ***Ceratocystis* sp. (SF17)** | 20±4 | 17±4 | 19.2±4 | 18.7±4 | 20±2 |
| ***Ceratocystis* sp. (DF18)** | 11.3±2 | 18.4±2 | 12.9±3 | 19.1±3 | 21±3 |
| *Ceratocystis* sp. (DF19) | 19±3 | 18.3±3 | 18.5±2 | 13±2 | 15±1 |
| *Ceratocystis* sp. (FF20) | 11±4 | 15±4 | 7.9±2 | 10.6±4 | 13.5±2 |
| *C.cerefabiensis* (QF21) | 10±3 | 13.7±2 | 11±3 | 14.3±1 | 15.6±3 |
| *C. corybiicola* (SF22) | 9.5±2 | 8.4±2 | 8.5±2 | 7.8±2 | 8.9±2 |
| ***C. corybiicola* (JF23)** | 14±1 | 21.5±2 | 22.5±2 | 17.3±3 | 19.5±2 |
| *C. corybiicola* (DF24) | 13.2±2 | 10±1 | 11±4 | 9.3±2 | 9.4±2 |
| *C. corybiicola* (DF25) | 10.8±2 | 9.7±3 | 13±1 | 10±2 | 9±2 |
| *C. corybiicola* (DF26) | 8.8±4 | 7.7±2 | 6.8±2 | 6.7±2 | 9.7±2 |
| *C. corybiicola* (DF27) | 7.9±3 | 12±1 | 17±3 | 7.6±3 | 8.6±2 |
| *C. corybiicola* (DF28) | 8.4±2 | 10±2 | 8.9±2 | 8.7±2 | 10.7±2 |
| *C. corybiicola* (RF29) | 8.9±3 | 9.3±2 | 7.6±2 | 8.9±2 | 7.4±2 |
| *C. corybiicola* (YF30) | 10±4 | 6.9±3 | 9.9±2 | 8.6±3 | 8.2±2 |
| *C. corybiicola* (DF31) | 7.4±4 | 8.6±4 | 6.9±1 | 6.7±2 | 8.2±3 |
| *C. corybiicola* (DF32) | 8.3±2 | 7.3±2 | 6.8±2 | 8.4±3 | 9.5±4 |
| *C. polychorma* (AF33) | 13±2 | 14.3±2 | 13±2 | 16±3 | 14±2 |
| *C. polychorma* (JF34) | 19±1 | 18±4 | 16.7±3 | 18.7±2 | 16.9±2 |
| *Cladosporium* sp. (RF35) | 12.3±3 | 2.9±3 | 10±2 | 2.4±4 | 11±1 |
| *Cladosporium* sp. (SF36) | 4±2 | 3.5±2 | 4.5±2 | 2±4 | 10.5±2 |
| *Cladosporium* sp. (JF37) | 9±2 | 5±2 | 8±1 | 4±2 | 9.5±2 |
| *Cladosporium* sp. (RAF38) | 7.8±3 | 9.7±2 | 13±2 | 10±2 | 9±2 |
| *Cladosporium* sp. (FF39) | 6.8±2 | 10±1 | 9.6±4 | 9.9±2 | 11±2 |
| ***Cladosporium* sp. (JEF40)** | 16.6±4 | 9.7±2 | 20±4 | 15±2 | 18±2 |
| *Cladosporium* sp. (JEF41) | 15±2 | 10±2 | 18±2 | 13±2 | 18±1 |
| *Cladosporium* sp. (JEF42) | 15.9±2 | 12±2 | 16±2 | 8.9±1 | 16.5±3 |
| *Cladosporium* sp. (JEF43) | 13±3 | 13±4 | 16.5±2 | 10±3 | 17±3 |
| *Cladosporium* sp. (JEF44) | 6.6±1 | 14±3 | 18.9±2 | 9.2±3 | 15±3 |
| *Cladosporium* sp. (SF45) | 9±2 | 12.9±2 | 12.4±2 | 17.8±2 | 18.6±3 |
| *Cladosporium* sp. (SF46) | 8.7±2 | 11.5±2 | 17.9±4 | 15.4±1 | 17±2 |
| *Cladosporium* sp. (HF47) | 11±2 | 12±2 | 15±3 | 21±2 | 16.9±2 |
| *Cladosporioides* (JF48) | 11±2 | 14±3 | 10.2±2 | 8.7±3 | 13.6±2 |
| *C.cladosporioids* (RAF49) | 12±3 | 11.3±4 | 16.8±2 | 11.3±4 | 10±3 |
| *C.cladosporioids* (FF50) | 13±2 | 13.2±2 | 11±3 | 10.9±3 | 11±3 |
| *C.cladosporioids* (QF51) | 12±2 | 14±1 | 10±2 | 9.8±2 | 16±4 |
| *C.cladosporioids* (JEF52) | 13±2 | 14.3±2 | 8±2 | 9.3±3 | 10.2±2 |
| *C.cladosporioids* (JEF53) | 11±2 | 18±2 | 10±2 | 19±4 | 10±1 |
| *C.cladosporioids* (JEF54) | 12±1 | 17±1 | 11.3±3 | 11±1 | 10±2 |
| ***C.oxysporum* (JEF55)** | 20±1 | 18±1 | 20±2 | 12±1 | 12±3 |
| ***C.oxysporum* (UF56)** | 19±3 | 21±3 | 20±4 | 8.7±2 | 13±1 |
| ***C.oxysporum* (JF57)** | 19.4±2 | 23.7±4 | 20.3±4 | 12.1±2 | 13±2 |
| ***C.oxysporum* (JF58)** | 18.3±4 | 19±2 | 21±3 | 8±4 | 16±3 |
| ***C.oxysporum* (JF59)** | 19.6±2 | 18±2 | 20.9±4 | 12±2 | 15±4 |
| *C.oxysporum* (JUF60) | 19±3 | 18±4 | 19.4±4 | 7.8±2 | 8.6±2 |
| *C. perangustum* (JEF61) | 10.3±2 | 5.2±3 | 3.9±2 | 10.2±4 | 5.3±2 |
| *C. perangustum* (JEF62) | 3.9±2 | 9±4 | 10.3±3 | 12±2 | 3.1±3 |
| *C. perangustum* (JEF63) | 10.3±2 | 2.6±4 | 9.7±3 | 2.4±1 | 10±4 |
| *C. tenuissium* (KF64) | 6.7±3 | 8.5±2 | 3.9±2 | 12.4±1 | 7±2 |
| *C. tenuissium* (JEF65) | 11±4 | 15±2 | 16.5±2 | 10±2 | 3.9±4 |
| *C. tenuissium* (UF66) | 9±2 | 2.4±1 | 9.3±1 | 8.1±3 | 10.2±2 |
| *C.tenuissium* (JEF67) | 4.6±2 | 10.3±3 | 11.6±3 | 9±2 | 11.2±4 |
| *C.tenuissium* (FF68) | 9.2±2 | 11±1 | 12.8±3 | 9.8±2 | 13.4±3 |
| ***D. hubeiensis*(UF69)** | 23±2 | 22.4±3 | 21.3±1 | 22.1±2 | 20.2±2 |
| *Emericellopsis* sp.(QF70) | 12.2±2 | 9.5±2 | 7.6±2 | 11±3 | 9±3 |
| *Emericellopsis* sp.(AF71) | 16±2 | 9.7±1 | 8.5±2 | 3.6±3 | 18±3 |
| *Emericellopsis* sp.(JF72) | 10.2±2 | 19±2 | 13.2±2 | 12.2±4 | 19±2 |
| ***Emericellopsis* sp.(FF73)** | 20±4 | 9.5±1 | 18±3 | 15±2 | 20±2 |
| ***Emericellopsis* sp.(FF74)** | 21.5±4 | 9.7±4 | 19.5±2 | 15±2 | 21±3 |
| ***Emericellopsis* sp.(FF75)** | 20-±2 | 9.8±3 | 19±2 | 10±2 | 20±4 |
| *E. alkaline*(FF76) | 10±4 | 9.7±2 | 4.3±1 | 2.9±2 | 10.2±2 |
| ***E. alkaline*(FF77)** | 19±3 | 23±2 | 17±0.2 | 21±0.1 | 18±0.3 |
| ***E. alkaline*(JF78)** | 20±3 | 20±3 | 24±3 | 20±1 | 22±3 |
| ***E. alkaline*(RAF79)** | 21±2 | 19±1 | 16±2 | 19±3 | 17±1 |
| ***E. alkaline*(FF80)** | 19.9±3 | 18±2 | 21±2 | 20.9±0.1 | 18±2 |
| *E.phycophila*(AF81) | 9.2±2 | 4.2±4 | 19.2±3 | 10±0.2 | 5.2±3 |
| *E.phycophila*(HF82) | 11.9±1 | 12.3±3 | 14.3±4 | 14.9±4 | 13.9±4 |
| *E.phycophila*(DF83) | 8.7±2 | 7.2±3 | 9.3±2 | 8.9±2 | 3.2±1 |
| *F. magnifereae*(JEF84) | 12±2 | 12±2 | 9.4±2 | 8.8±3 | 6.6±3 |
| *H. alpina*(AF85) | 4.6±2 | 10.3±1 | 8.6±4 | 9±2 | 6.2±2 |
| *L. theobromae*(DF86) | 7±2 | 9.4±2 | 6.3±1 | 5.1±3 | 11.2±2 |
| ***Microdochium* sp.(SF87)** | 22.3±4 | 22.9±2 | 15±2 | 22.4±2 | 18±2 |
| ***Microdochium* sp.(HF88)** | 24±3 | 23.5±1 | 24.5±2 | 23±2 | 20.5±3 |
| ***Microdochium* sp.(HF89)** | 20±1 | 25±2 | 21±2 | 24±3 | 21.5±3 |
| *M.anisopliae*(SF90) | 8.2±2 | 1.1±2 | 2.8±3 | 3.8±2 | 3.4±2 |
| *Nannizzia* sp.(DF91) | 3.2±3 | 4.8±3 | 8.3±1 | 8.6±1 | 9.2±2 |
| *Nannizzia* sp.(JUF92) | 6.7±2 | 9±2 | 10±2 | 8±1 | 11±1 |
| *N. gypsea*(HF93) | 6±4 | 8±2 | 8.3±2 | 6.4±1 | 7.9±1 |
| ***P. glabrae*(YF94)** | 21±2 | 25.8±2 | 23.5±3 | 20±2 | 23.9±2 |
| *Sordariyomycetes* sp.(RF95) | 16.7±1 | 8.5±1 | 13.9±2 | 12.4±2 | 17±1 |
| *S. glycines*(SF96) | 3.6±3 | 5.3±3 | 6.6±3 | 5±1 | 2.2±4 |
| *Usnea* sp*.*(FF97) | 9.9±1 | 10.5±2 | 11±2 | 8.9±1 | 10.8±4 |
| *Usnea* sp*.*(FF98) | 8.9±3 | 12±1 | 9.2±3 | 8.3±2 | 7.9±3 |
| *Usnea* sp*.*(AF99) | 11±2 | 7.9±3 | 6.9±3 | 8.5±2 | 11±2 |
| *U.cornuta*(RF100) | 9.8±3 | 11±2 | 10.9±1 | 13±2 | 12±1 |
