# Supplementary material for: Marine fungi showing multifunctional activity against human pathogenic microbes and cancer
Source: PLoS One. 2022 Nov 28;17(11):e0276926. doi: 10.1371/journal.pone.0276926 (PMC9704632; doi:10.1371/journal.pone.0276926)
Supplement: S2 Table — Mean ≥ 20 mm against some fungal pathogens in bold. (DOCX) [file pone.0276926.s002.docx]

**Marine fungi producing potential multifunctional components against pathogenic microbes and cancer**

Fuad Ameen^1*^, Saleh AlNAdhari^2^ and Ali A. Al-Homaidan^1^

^1^Department of Botany and Microbiology, College of Science, King Saud University, Riyadh 11451, Saudi Arabia

^2^Deanship of Scientific Research, King Saud University, Riyadh 11451, Saudi Arabia

Correspondence to: [fuadameen@ksu.edu.sa](mailto:fuadameen@ksu.edu.sa)

**S2 Table:** Inhibition (zone mm, mean ± SD, *n* = 3) of five pathogenic fungi by fungicides (Gentamycin and Fluconazole, 5 μg/mL) and marine fungal extracts. Mean ≥ 20 mm against some fungal pathogens in bold.

| Fungal inhibitor | *Aspergillus fumigatus* | *Candida albicans* | *Cryptococcus neoformans* | *Malassezia globosa* | *Talaromyces marneffei* |
| --- | --- | --- | --- | --- | --- |
| **Gentamycin** | **20±0.1** | **24.4±0.1** | **21±0.1** | **23±0.1** | **22±0.1** |
| **Fluconazole** | **21.5±0.1** | **21±0.1** | **23±0.1** | **22±0.1** | **20±0.1** |
| ***Acremonium* sp.(JEF2)** | 22±2 | 22±3 | 22±2 | 28±3 | 26±4 |
| ***Acremonium* sp.(JEF1)** | 22±2 | 20.5±1 | 19±2 | 23.6±3 | 22±3 |
| ***Acremonium* sp.(SF3)** | 20.7±1 | 21±2 | 19.9±1 | 24±2 | 26±3 |
| ***Acrocalymma* sp.(JF4)** | 25±2 | 21.8±2 | 10.3±2 | 21±4 | 13.1±1 |
| ***A. africana*(SF5)** | 24±3 | 20.2±3 | 11.9±3 | 22±2 | 14.3±2 |
| ***A. medicaginis*(RAF6)** | 27±4 | 21±1 | 12.7±2 | 21.4±3 | 10±2 |
| *Aspergillus* sp.(HF7) | 0.2±3 | 2.1±2 | 2.8±1 | 11.8±2 | 1±4 |
| *Aspergillus* sp.(DF8) | 0.3±2 | 0.2±2 | 2.9±3 | 12±1 | 1.3±2 |
| *Aspergillus* sp.(JEF9) | 0.9±1 | 1.8±1 | 2.3±2 | 12±2 | 1.1±3 |
| *Buellia* sp. (HF10) | 2.6±2 | 1.8±3 | 1.9±2 | 2.6±1 | 2.5±3 |
| *B. lauricassiae*(HF11) | 1.6±3 | 1.9±4 | 2.3±4 | 2.2±3 | 1.8±3 |
| *B. lauricassiae*(HF12) | 1.6±2 | 2.0±2 | 1.9±2 | 1.9±2 | 3.1±3 |
| *Ceratocystis* sp.(DF13) | 3.5±3 | 3.9±2 | 0.8±2 | 1.4±2 | 2.2±2 |
| *Ceratocystis* sp.(DF14) | 1.5±2 | 2.4±2 | 2.0±1 | 1.3±2 | 2.0±2 |
| *Ceratocystis* sp.(JUF15) | 1.7±2 | 1.4±3 | 1±3 | 1.9±3 | 1.9±1 |
| *Ceratocystis* sp.(KF16) | 1.8±3 | 0.2±4 | 4.6±1 | 1.4±2 | 2.1±2 |
| *Ceratocystis* sp.(SF17) | 2.0±4 | 1.7±2 | 3.2±2 | 2.7±2 | 2.0±2 |
| *Ceratocystis* sp.(DF18) | 1.3±2 | 1.4±2 | 2.9±4 | 2.1±4 | 2.1±3 |
| *Ceratocystis* sp.(DF19) | 1.9±2 | 1.3±3 | 1.8±1 | 3±1 | 1.5±3 |
| *Ceratocystis* sp.(FF20) | 1.8±2 | 1.5±2 | 2.9±3 | 1.6±2 | 1.3±4 |
| *C.cerefabiensis*(QF21) | 3.0±3 | 1.3±4 | 1.1±2 | 1.3±4 | 1.6±2 |
| *C. Corybiicola* (SF22) | 1.5±2 | 2.4±2 | 4.5±2 | 0.8±2 | 0.9±2 |
| *C. Corybiicola* (JF23) | 1.4±3 | 1.5±4 | 2.2±3 | 1.7±2 | 1.9±2 |
| *C. corybiicola*(DF24) | 1.3±2 | 1.0±2 | 1.1±1 | 0.3±1 | 0.4±2 |
| *C. corybiicola*(DF25) | 0.8±2 | 1.7±3 | 1.3±2 | 1.0±2 | 0.4±3 |
| *C. corybiicola*(DF26) | 0.8±2 | 0.7±4 | 0.8±3 | 0.7±2 | 1.7±2 |
| *C. corybiicola*(DF27) | 0.9±2 | 1.2±1 | 1.7±2 | 1.6±3 | 1.6±1 |
| *C. corybiicola*(DF28) | 0.4±1 | 1.0±4 | 0.9±2 | 0.7±1 | 1.0±3 |
| *C. corybiicola*(RF29) | 0.9±3 | 0.3±1 | 0.6±2 | 0.9±3 | 0.4±2 |
| *C. corybiicola*(YF30) | 1.0±3 | 0.9±2 | 0.9±2 | 1.6±4 | 0.2±3 |
| *C. corybiicola*(DF31) | 0.4±4 | 0.6±3 | 1.9±2 | 0.7±2 | 1.2±2 |
| *C. corybiicola*(DF32) | 0.3±2 | 1.3±4 | 0.8±2 | 0.4±2 | 0.5±1 |
| *C.polychorma*(AF33) | 1.3±1 | 1.3±3 | 1.3±3 | 1.6±1 | 1.4±1 |
| *C.polychorma*(JF34) | 1.9±1 | 1.8±4 | 1.7±3 | 1.7±2 | 1.6±4 |
| *Cladosporium* sp.(RF35) | 10±1 | 8.9±1 | 10.5±3 | 8.4±1 | 10±3 |
| *Cladosporium* sp.(SF36) | 12±1 | 9.5±1 | 10.5±3 | 8±1 | 11±2 |
| *Cladosporium* sp.(JF37) | 9.9±4 | 11±1 | 11±2 | 9±2 | 9.8±2 |
| *Cladosporium* sp.(RAF38) | 10.8±4 | 10.7±1 | 10±2 | 11±3 | 9.9±4 |
| *Cladosporium* sp.(FF39) | 10.8±4 | 11±2 | 11.6±4 | 1.9±4 | 10±3 |
| *Cladosporium* sp.(JEF40) | 9.6±2 | 9.7±3 | 11±4 | 10±2 | 11±2 |
| *Cladosporium* sp.(JEF41) | 9.8±2 | 11±2 | 10±3 | 11±2 | 10±2 |
| *Cladosporium* sp.(JEF42) | 10.9±4 | 9±3 | 10±2 | 10.9±3 | 11.5±3 |
| *Cladosporium* sp.(JEF43) | 10±1 | 10±2 | 11±2 | 10±2 | 11±3 |
| *Cladosporium* sp.(JEF44) | 9.6±2 | 10±2 | 11±3 | 9.2±3 | 11±4 |
| *Cladosporium* sp.(SF45) | 10±2 | 9.9±2 | 10.4±1 | 10±3 | 9.6±4 |
| *Cladosporium* sp.(SF46) | 10.7±3 | 9.5±3 | 10±2 | 10±2 | 8.9±2 |
| *Cladosporium* sp.(HF47) | 11±2 | 10±3 | 15±2 | 11±3 | 9.9±2 |
| *C.cladosporioids*(JF48) | 10±2 | 12±2 | 10±3 | 9.7±2 | 11.5±2 |
| *C.cladosporioids*(RAF49) | 11±2 | 10.3±4 | 12±2 | 12.3±1 | 10±2 |
| *C.cladosporioids*(FF50) | 12±3 | 11.2±2 | 10±2 | 10±3 | 12±3 |
| *C.cladosporioids*(QF51) | 12±4 | 10±2 | 11±2 | 11±2 | 10±2 |
| *C.cladosporioids*(JEF52) | 10±2 | 11.3±2 | 9±2 | 10±2 | 11±1 |
| *C.cladosporioids*(JEF53) | 12.5±2 | 13±1 | 11±2 | 12±3 | 12±2 |
| *C.cladosporioids*(JEF54) | 10±2 | 11±1 | 12±4 | 10±4 | 11±1 |
| *C.oxysporium*(JEF55) | 12±2 | 12±2 | 11±2 | 10.3±2 | 9±3 |
| *C.oxysporium*(UF56) | 14±3 | 12±2 | 10±1 | 9.7±3 | 11±4 |
| *C.oxysporium*(JF57) | 12.4±4 | 13±3 | 12.3±2 | 11±2 | 13±1 |
| *C.oxysporium*(JF58) | 11.3±1 | 10±3 | 9±4 | 11±2 | 11±2 |
| *C.oxysporium*(JF59) | 10.6±1 | 11±2 | 9.9±2 | 10±4 | 11±2 |
| *C.oxysporium*(JUF60) | 11±2 | 10±1 | 9.4±4 | 8.8±1 | 10.6±3 |
| *C.perangustum*(JEF61) | 10.3±2 | 9.2±2 | 9.9±2 | 9±3 | 9.3±4 |
| *C.perangustum*(JEF62) | 13.9±2 | 10±4 | 10.3±3 | 10±3 | 10±4 |
| *C.perangustum*(JEF63) | 11.3±3 | 10.6±3 | 9.7±2 | 9.4±4 | 11±2 |
| *C.tenuissium*(KF64) | 8.7±4 | 8±2 | 8.9±4 | 10.4±2 | 9±1 |
| *C.tenuissium*(JEF65) | 10±4 | 10±1 | 10.5±2 | 10±4 | 9.9±2 |
| *C.tenuissium*(UF66) | 11±1 | 9.4±2 | 9.8±3 | 8.3±1 | 10.2±3 |
| *C.tenuissium*(JEF67) | 10±1 | 10±3 | 10.6±4 | 8.5±4 | 10.2±2 |
| *C.tenuissium*(FF68) | 9.8±2 | 10±3 | 11.8±2 | 9.2±2 | 11.4±4 |
| ***D. hubeiensis*(UF69)** | 22±3 | 24±2 | 17±3 | 20±2 | 23.2±3 |
| *Emericellopsis* sp.(QF70) | 12±3 | 13.5±3 | 12.6±2 | 18±4 | 10±4 |
| *Emericellopsis* sp.(AF71) | 14±2 | 11.7±2 | 11.5±1 | 18.6±3 | 13±2 |
| *Emericellopsis* sp.(JF72) | 10±4 | 11±2 | 13.8±1 | 17.2±1 | 14±2 |
| *Emericellopsis* sp.(FF73) | 10±4 | 14.5±3 | 14±2 | 17±2 | 12±3 |
| *Emericellopsis* sp.(FF74) | 11.5±2 | 14.7±1 | 12.5±1 | 15±3 | 10±2 |
| *Emericellopsis* sp.(FF75) | 14±1 | 15.8±2 | 12±2 | 18±2 | 13±1 |
| *E. alkalina*(FF76) | 10±1 | 8.7±1 | 7.3±1 | 9.9±1 | 11.2±1 |
| *E. alkalina*(FF77) | 11.6±3 | 12.7±3 | 15±2 | 11±2 | 10.4±3 |
| *E. alkalina*(JF78) | 10±2 | 10±4 | 13.6±2 | 10.7±1 | 11±4 |
| *E. alkalina*(RAF79) | 12.2±3 | 9.3±2 | 12±3 | 10±2 | 10.6±2 |
| *E. alkalina*(FF80) | 7.9±2 | 8±2 | 8.1±2 | 2.9±4 | 2.2±2 |
| *E.phycophila*(AF81) | 15.2±1 | 14.2±2 | 16.2±2 | 10±2 | 5.2±2 |
| *E.phycophila*(HF82) | 13.9±4 | 15.3±3 | 14.8±4 | 14±2 | 13.9±1 |
| *E.phycophila*(DF83) | 10±1 | 11±2 | 1.4±3 | 5.8±2 | 1.6±1 |
| *F. magnifereae*(JEF84) | 0.6±2 | 0.3±2 | 0.6±4 | 1.9±4 | 0.2±1 |
| *H. alpina*(AF85) | 3±2 | 1.4±2 | 0.4±2 | 0.6±4 | 10.2±2 |
| ***L. theobrome*(DF86)** | 22±3 | 23±1 | 14±2 | 22±3 | 19±3 |
| ***Microdochium* sp.(SF87)** | 25±3 | 24±3 | 11.5±4 | 21±3 | 18±1 |
| ***Microdochium* sp.(HF88)** | 24±2 | 25±2 | 14±2 | 24±2 | 19±2 |
| *Microdochium* sp.(HF89) | 1.2±4 | 0.1±4 | 4.8±2 | 0.8±2 | 0.4±2 |
| *M.anisopliae*(SF90) | 0.2±1 | 0.8±4 | 0.3±4 | 0.6±2 | 0.2±4 |
| *Nannizzia* sp.(DF91) | 0.7±2 | 0.5±4 | 0.8±2 | 0.8±1 | 0.1±4 |
| *Nannizzia* sp.(JUF92) | 0.6±3 | 0.8±2 | 0.3±3 | 0.4±1 | 0.9±1 |
| ***N. gypsea*(HF93)** | 25±2 | 27±1 | 18.5±3 | 25±2 | 23±1 |
| *P. glabrae*(YF94) | 1.7±1 | 0.5±4 | 10.9±2 | 1.2±2 | 1.7±2 |
| *Sordariyomycetes* sp.(RF95) | 0.6±2 | 1.3±2 | 1.6±1 | 2±0.1 | 3.2±2 |
| *S. glycines*(SF96) | 2.9±3 | 1.0±3 | 1.1±2 | 0.9±2 | 1.8±3 |
| *Usnea* sp*.*(FF97) | 2.9±3 | 1.0±1 | 1.1±2 | 0.9±2 | 1.8±2 |
| *Usnea* sp*.*(FF98) | 1.9±4 | 1.2±2 | 1.2±3 | 0.3±2 | 0.9±3 |
| *Usnea* sp*.*(AF99) | 1.1±2 | 1.9±2 | 0.9±3 | 0.5±1 | 1.1±2 |
| *U.cornuta*(RF100) | 2.8±1 | 1.1±2 | 1.9±2 | 0.3±1 | 1.2±2 |
