# Supplementary material for: Marine fungi showing multifunctional activity against human pathogenic microbes and cancer
Source: PLoS One. 2022 Nov 28;17(11):e0276926. doi: 10.1371/journal.pone.0276926 (PMC9704632; doi:10.1371/journal.pone.0276926)
Supplement: S3 Table — (DOC) [file pone.0276926.s003.doc]

**Marine fungi producing potential multifunctional components against pathogenic microbes and cancer**

Fuad Ameen^1*^, Saleh AlNAdhari^2^ and Ali A. Al-Homaidan^1^

^1^Department of Botany and Microbiology, College of Science, King Saud University, Riyadh 11451, Saudi Arabia

^2^Deanship of Scientific Research, King Saud University, Riyadh 11451, Saudi Arabia

Correspondence to: fuadameen@ksu.edu.sa

**S3 Table**. Variable loadings of PCA for different cancer types, bacterial and fungal pathogens and antioxidant activity assays.

| PC1 | PC2 | Variable |
| --- | --- | --- |
| 0.80 | -0.40 | Liver |
| 0.81 | -0.37 | Skin |
| 0.80 | -0.36 | Breast |
| 0.79 | -0.36 | Lung |
| 0.73 | -0.36 | *Bacillus subtilis* |
| 0.66 | -0.33 | *Salmonella typhi* |
| 0.75 | -0.28 | *S. paratyphi* |
| 0.76 | -0.27 | *Staphylococcus aureus* |
| 0.18 | 0.00 | *Vibrio cholerae* |
| 0.74 | 0.61 | *Malassezia globosa* |
| 0.61 | 0.70 | *Cryptococcus neoformans* |
| 0.64 | 0.72 | *Aspergillus fumigatus* |
| 0.61 | 0.73 | *Talaromycets marneffei* |
| 0.64 | 0.74 | *Candida albicans* |
| 0.85 | -0.25 | DPPH |
| -0.25 | -0.14 | Reducing power |
